# Prävention von Ballsportunfällen an Schulen mittels eines „serious game“: Konzeptvorstellung und begleitende Evaluation

**DOI:** 10.1007/s11553-021-00886-1

**Published:** 2021-07-23

**Authors:** Valentin Riemer, Claudia Schrader, Olga Pollatos

**Affiliations:** 1 Fakultät Ingenieurwissenschaften, Informatik und Psychologie, Institut für Psychologie und Pädagogik, Universität Ulm, Albert-Einstein-Allee 47, 89081 Ulm, Deutschland; 2 Institut für Bildungsforschung in de r School of Education, Bergische Universität Wuppertal, Wuppertal, Deutschland

**Keywords:** Verletzungsprävention, Schulsport, Ballspiel, Lernspiel, „Blended learning“, Injury prevention, School sports, Ball game, Game-based learning, Blended learning

## Abstract

**Hintergrund:**

Die Zahl der Ballsportunfälle im Schulsport ist seit Jahren konstant hoch. Zu den Hauptursachen zählt mangelnde Gefahrenkenntnis und eine daraus resultierende geringe Risikowahrnehmung bei Schülerinnen und Schülern (SuS).

**Ziel der Arbeit:**

Ein Präventionskonzept wird vorgestellt, bestehend aus einem „serious game“ und didaktischen Begleitmaterialien für SuS und Lehrkräfte. Lernziele, Spielprinzipien und Anwendung in einem Blended-learning-Ansatz werden beschrieben. Zwei begleitende Evaluationsstudien und deren wichtigsten Ergebnisse werden berichtet.

**Material und Methoden:**

Mittels teilstrukturierter Interviews wurden in Studie 1 (*n*_SuS_ = 92; *n*_Lehrkräfte_ = 9) Vorwissen und Erwartungen der Zielgruppen erhoben. Nach Entwicklung eines Spielprototyps wurde dieser in einer Studie 2 (*n*_SuS_ = 13; *n*_Lehrkräfte_ = 8; *n*_Expert*innen_ = 5) eingesetzt und mittels Online-Fragebogen das Spielerleben sowie Einstellungen zur Nutzung des Präventionskonzepts erfasst.

**Ergebnisse:**

Die SuS zeigten nur geringes Vorwissen zu Gefahren im Ballsport an Schulen. SuS berichteten ein generell positives Spielerleben. Lehrkräfte zeigten eine positive Einstellung zum Präventionskonzept.

**Diskussion:**

Die Erkenntnisse aus den Studien flossen im Sinne eines Design-based-research-Ansatzes direkt in die weitere Entwicklung ein. Es wird eine hohe Anwendungsbereitschaft für das Präventionskonzept angenommen. Die Effektivität des Konzepts ist in einer abschließenden Evaluationsstudie zu prüfen.

## Abstract (en)

**Background:**

The number of ball game injuries at school has remained on a high level over the recent years. The main causes are considered to be little knowledge about potential hazards and, as a result, low risk awareness among pupils.

**Objective:**

We introduce a prevention concept, comprising a serious game and accompanying didactical materials for pupils and teachers. We describe the learning goals, game mechanics and the practical application of the concept in a blended-learning setting. In addition, relevant results of two studies in the course of a concomitant evaluation are reported.

**Materials and methods:**

In study 1, prior knowledge and expectations of the target groups were assessed in semistructured interviews (*n*_Pupils_ = 92; *n*_Teachers_ = 9). In study 2, following the development of a game prototype, gameplay experience and attitudes towards using the concept were collected in an online survey (*n*_Pupils_ = 13; *n*_Teachers_ = 8; *n*_Experts_ = 5).

**Results:**

Pupils had little knowledge about hazards in ball games at schools. Pupils reported their gameplay experience to be generally positive. Teachers reported generally positive attitudes towards the concept.

**Discussion:**

Results from both studies guided the further development of the game and accompanying materials. We expect a high readiness of the target groups to use the concept. The effectivity of the concept is yet to be tested in a concluding evaluation.

## Einleitung

Ballsportarten zählen nicht nur zu den populärsten Sportarten bei Schülerinnen und Schülern (SuS), sondern auch zu den risikoreichsten in Hinblick auf Verletzungen im Schulsport in Deutschland. In den Berichten der Deutschen Gesetzlichen Unfallversicherung e. V. (DGUV) zum schulischen Unfallgeschehen der letzten Jahre entfallen konstant mehr als die Hälfte aller Unfälle im Schulsport auf den Ballsport [2, 3]. Als einer der Hauptgründe dafür wird häufig eine unzureichende Gefahrenkenntnis der SuS genannt [13, 17]. Dies beinhaltet mangelndes Wissen über Gefahrensituationen und Unfallmechanismen im Ballsport sowie über daraus resultierende mögliche Verletzungen. In weiterer Folge kann dies zu einem verringerten wahrgenommenen Risiko und damit einer erhöhten Unfallgefahr führen [17].

Neben der Gefahrenkenntnis spielt zudem die Motivation zu sicherem Verhalten und zur Auseinandersetzung mit Unfallprävention eine entscheidende Rolle [13]. Motivation ist entscheidend für die Aufnahme erwünschter Verhaltensweisen und zugleich abhängig von deren Übereinstimmung mit persönlichen Interessen und Zielen [12]. Besonders Jugendliche im Altersbereich von 12 bis 16 Jahren neigen jedoch zu riskanten Verhaltensweisen, da sicherheitsbezogenes Verhalten von anderen Zielen verdrängt wird, die den eigenen Werten näher stehen [16]. Daraus ergibt sich die erhöhte Notwendigkeit der Etablierung von gesundheitsbewussten Verhaltensweisen in dieser Altersgruppe, wie sie in anderen Bereichen der Prävention, wie etwa dem Tabakkonsum [14], bereits erfolgt.

Aus den genannten Gründen wurde ein Präventionskonzept entwickelt, mit dem Ziel, SuS im Jugendalter Gefahrenkenntnis im Ballsport zu vermitteln und zur Aufnahme sicheren Verhaltens im Schulsport zu motivieren. Im Zentrum des Konzepts steht dabei ein „serious game“, also ein digitales Lernspiel, dessen Zweck über die reine Unterhaltung hinausgeht. „Serious games“ sind in der Lage, komplexe Sachverhalte durch eine authentische und interaktive Erfahrung zu vermitteln [15]. Die Effektivität von „serious games“ in Bezug auf Prävention und Gesundheitsförderung konnte dabei bereits in den Bereichen Ernährung und körperliche Aktivität gezeigt werden [18].

Im Folgenden werden das „serious game“ *Basketball Training Manager*, dessen kognitive und motivationale Lernziele sowie die geplante Anwendung im Rahmen eines Blended-learning-Ansatzes beschrieben. Anschließend werden 2 Studien der begleitenden Konzeptevaluation vorgestellt, deren Ergebnisse in den Entwicklungsprozess einflossen. Abschließend werden die Erkenntnisse in Hinblick auf die geplante Anwendung diskutiert sowie ein Ausblick auf eine abschließende Evaluationsstudie gegeben.

## Das „serious game“ *Basketball Training Manager*

Das Spiel wurde unter Leitung der Universität Ulm entwickelt und von der Firma Gentle Troll Entertainment GmbH realisiert. Es handelt sich dabei um eine im Web-Browser spielbare Sportsimulation.

Der inhaltliche Fokus auf Basketball als eines der vier „großen Ballspiele“ (mit Fußball, Handball und Volleyball) wurde aufgrund mehrerer Faktoren gewählt. So zählt Basketball sowohl zu den meist gespielten Ballsportarten an Schulen als auch zu den in Relation zur Expositionszeit risikoreichsten [7]. Darüber hinaus sind Verletzungen im Basketball im Schulsport bei Jungen und Mädchen annähernd gleich verteilt [4]. Schließlich ähneln sowohl Unfallsituationen als auch Verletzungen im Basketball jenen in Handball und Volleyball [11], wodurch die vermittelten Inhalte für ein breites Spektrum an Ballsportarten gültig sind.

### Lernziele

Die kognitiven Lernziele des *Basketball Training Managers* umfassen erstens die Vermittlung des Wissens zu Unfallmechanismen sowie zu Verletzungen im Ballsport an Schulen. Zweitens sollen SuS lernen, die Zusammenhänge der Unfallmechanismen mit den protektiven Faktoren koordinative Fähigkeiten, Antizipationsfähigkeit und Konzentration zu verstehen.

Hinsichtlich Unfallmechanismen ist für Basketball, Handball und Volleyball zunächst die individuelle Ballbehandlung (insbesondere das Passen und Fangen) als die mit Abstand häufigste Unfallsituation zu nennen [11]. Dahinter folgt bei allen drei Ballsportarten das Laufen bzw. Stürzen mit und ohne Ball. Entsprechend sind vor allem die oberen Extremitäten und hier besonders die Finger und Hände von Verletzungen betroffen [11].

Ein weiterer, sportartunabhängiger Unfallmechanismus betrifft die Vertrautheit von Bewegungen [17]. Etwa 60 % der Unfälle im Schulsport ereignen sich bei bereits oft durchgeführten Bewegungen unter variablen Bedingungen. Auch bei simpler Wiederholung einer bereits bekannten Bewegung ereignen sich etwa 20 % der Unfälle. Auf das Erlernen einer neuen Bewegung entfallen hingegen lediglich 7 % aller Unfälle [9]. Als Ursachen hierfür sind die Unterschätzung der Gefahrensituation bzw. die Überschätzung der eigenen Fertigkeit sowie ein Abfall der Konzentration und erhöhte Ablenkbarkeit zu nennen [10].

Als protektive Faktoren, die in Zusammenhang mit den genannten Unfallmechanismen stehen, sind zunächst koordinative Fähigkeiten zu nennen. Diese sind für die sichere Ausführung von Bewegungen im Schulsport unerlässlich [6].

Darüber hinaus können Antizipation und Konzentration als protektive Faktoren für Ballsportunfälle gezählt werden. Antizipation beschreibt dabei die Fähigkeit, die Bewegungen des Balls sowie der Mitspielenden vorweg zu nehmen und entsprechend zu reagieren [7]. Die Aufrechterhaltung von Konzentration schließlich ist wesentlich, um während Ballsporteinheiten nicht durch Ablenkung oder Flüchtigkeitsfehler in Gefahr einer Verletzung zu kommen [10].

Zusätzlich zu den kognitiven Lernzielen soll die Motivation zur Unfallprävention im Ballsport durch die Erhöhung der Übereinstimmung zwischen präventivem Verhalten und persönlichen Zielen der SuS gefördert werden [12].

### Spielprinzip

**Abb. 1:**
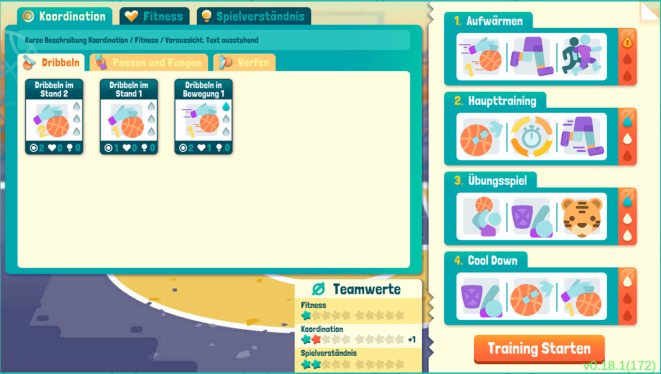
Trainingsplanerstellung: *links* zur Auswahl stehende Übungen nach Kategorien; *rechts* Zuordnung der Übungen zu Trainingseinheiten

Im* Basketball Training Manager *übernehmen SuS die Rolle des Captains eines Teams in einer Street-Basketball-Liga, wo sie gegen andere virtuelle Teams antreten. Der Fokus liegt dabei auf der Erstellung von Trainingsplänen, wofür den SuS eine Vielzahl an Trainingsübungen zur Verfügung stehen (Abb. 1). Diese steigern einerseits die koordinativen Fähigkeiten und Antizipation (im Spiel als „Spielverständnis“ bezeichnet), stellen andererseits aber auch Belastungen dar, die zu Verletzungen führen können. Verletzt sich ein*e Spieler*in des Teams, steht diese*r für den Zeitraum der Rekonvaleszenz nicht zur Verfügung. Die Herausforderung für die SuS besteht also darin, das Training so zu gestalten, dass sich ihre Teams verbessern, ohne durch verletzungsbedingte Ausfälle von Spieler*innen eine gute Platzierung in der Liga zu riskieren. Die Trainingseinheiten und Matches laufen dabei automatisiert ab, wobei die SuS Eingriffsmöglichkeiten in Form von Auswechslungen haben.

**Abb. 2:**
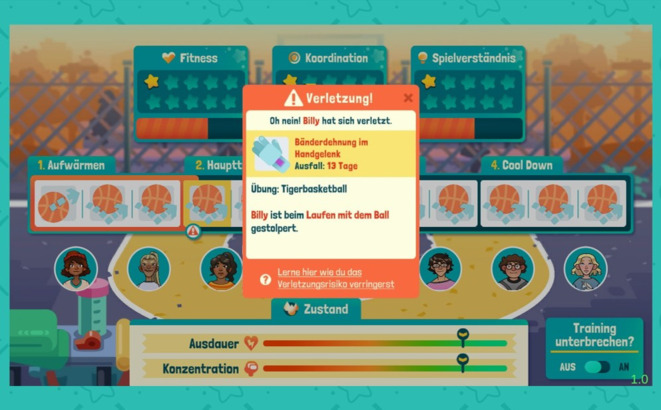
Verletzungsbenachrichtigung während eines Trainings

**Abb. 3:**
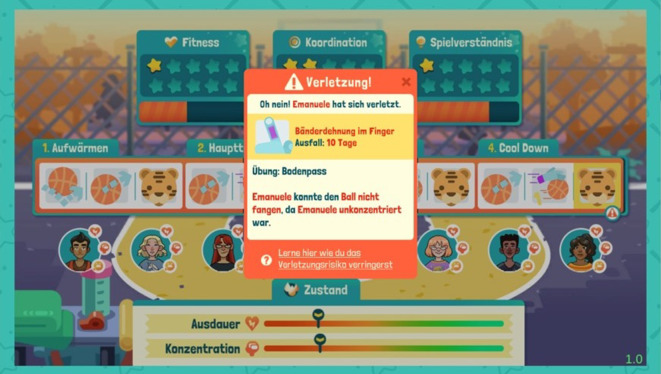
Verletzung aufgrund mangelnder Konzentration

**Abb. 4:**
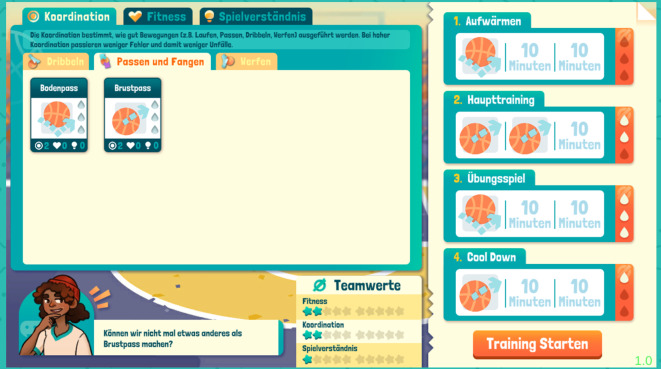
Trainingserstellung mit Hinweis auf zu viele Übungen aus einer Kategorie

Verletzungen können während der Trainingseinheiten und während der Spiele auftreten (Abb. 2). Die Auftrittswahrscheinlichkeiten für unterschiedliche Verletzungsarten orientieren sich an deren realen Prävalenzen im Schulsport und werden durch die Trainingsgestaltung beeinflusst. So fördern Trainingsübungen der Kategorien „Passen und Fangen“, „Dribbeln“ und „Werfen“ die koordinativen Fähigkeiten, während Übungen zu „Spielverständnis“ die Antizipation erhöhen. Durch entsprechendes Training verringert sich das Risiko, dass Unfallmechanismen, die mit den jeweiligen Faktoren in Zusammenhang stehen, ausgelöst werden. Andererseits verringert jede Übung gleichzeitig die Konzentration im Verlauf eines Trainings, wodurch sich das Risiko, entsprechende Unfallmechanismen auszulösen, erhöht (Abb. 3). Werden viele gleichartige Übungen in einer Trainingseinheit angesetzt, nimmt die Konzentration deutlich schneller ab. Damit wird das erhöhte Unfallrisiko bei oft wiederholten Bewegungen abgebildet. Um den SuS dies zu signalisieren, erhalten sie bei der Trainingserstellung einen Hinweis, wenn zu viele Übungen einer Kategorie gewählt wurden (Abb. 4).

**Abb. 5:**
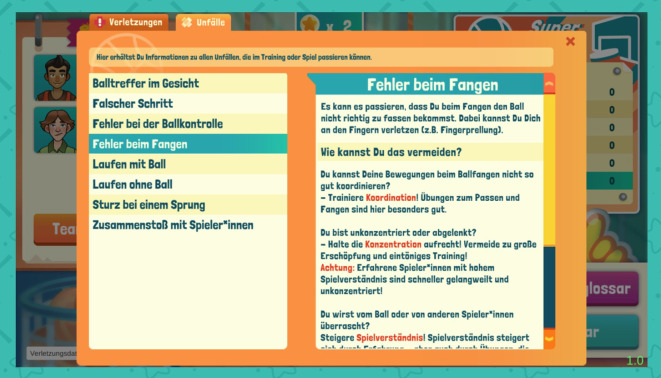
Unfallglossar mit Hinweisen zu Unfallmechanismen und Vorbeugung

Die Zusammenhänge zwischen Unfallmechanismen, Verletzungen und den protektiven Faktoren werden den SuS verdeutlicht, indem die relevanten Begriffe in den Benachrichtigungen zu den Unfällen farblich hervorgehoben werden (s. Abb. 3). Zusätzlich können die SuS von den Unfallbenachrichtigungen aus über einen Hyperlink auf Glossare zugreifen. Diese enthalten Beschreibungen von Unfallmechanismen, Verletzungen und Trainingsübungen sowie deren Verknüpfungen (Abb. 5). Die Glossare sind für die SuS außerdem jederzeit im Spiel aufrufbar.

Das motivationale Lernziel, betreffend die subjektive Bedeutung von Prävention, wird durch die Rolle der SuS als Captain des Teams angesprochen. Die SuS wählen zu Beginn des Spiels einen Avatar und können diesen mit dem eigenen Namen versehen. Als Captain ist der Spieleravatar selbst Teil des Teams und nimmt an Trainingseinheiten sowie Spielen teil. Dementsprechend besteht auch für den eigenen Avatar das Risiko, sich zu verletzen. Die Identifikation der SuS mit dem virtuellen Avatar kann somit die Übernahme gesundheitsrelevanter Verhaltensweisen begünstigen [5].

### Anwendung in einem Blended-learning-Ansatz

Die Einbindung des *Basketball Training Managers* in den Sportunterricht erfolgt mittels eines Blended-learning-Ansatzes. Dies erlaubt die Nutzung eines digitalen Lernformats ohne auf die Vorteile des Präsenzunterrichts zu verzichten und trägt dadurch zum Lerntransfer der im Spiel vermittelten Inhalte bei [1]. Die SuS spielen das Spiel dabei vornehmlich außerhalb der Unterrichtszeit, während im Präsenzunterricht die Inhalte aufgegriffen und reflektiert werden. So können etwa Trainingseinheiten, die im Spiel zusammengestellt wurden, im Sportunterricht umgesetzt und dadurch unterschiedliche Belastungsniveaus und Schwerpunktsetzungen erfahrbar gemacht werden. Hierzu werden den Lehrkräften didaktische Begleitmaterialien zur Verfügung gestellt. Diese enthalten, neben einem einführenden Überblick über Unfallmechanismen und Verletzungen im Ballsport an Schulen, eine Sammlung von 46 Übungen zur Förderung der koordinativen Fähigkeiten sowie der Antizipation für den Einsatz im Sportunterricht. Die Übungen wurden in Zusammenarbeit mit Experten aus der Sportpraxis ausgewählt und entsprechen jenen, die die SuS für die Trainingsgestaltung im Spiel zur Verfügung haben.

Die Begleitmaterialien enthalten darüber hinaus konkrete Anwendungsempfehlungen, etwa in Form von beispielhaften Unterrichtseinheiten. Diese sind nach Schwerpunkten und Schwierigkeitsgraden kategorisiert. Schließlich erhalten die Lehrkräfte in den Begleitmaterialien ein Tutorial zur Spielverwaltung. Über diese haben die Lehrkräfte die Möglichkeit, Spielstatistiken zu Spielfortschritt und Zahl der verletzten Spieler*innen der SuS einzusehen. Diese Informationen können die Reflexion im Präsenzunterricht unterstützen.

## Konzeptevaluation

Im Zuge einer begleitenden Evaluation wurden bereits im Vorfeld und während der Spielentwicklung Studien mit Lehrkräften, SuS sowie Expert*innen aus den Bereichen Lehr-Lernforschung sowie Mensch-Computer-Interaktion durchgeführt. Dies sollte den Praxisbezug des Präventionskonzepts sicherstellen sowie frühzeitig Ansätze zur Optimierung der Maßnahme aufzeigen.

### Studie 1 – Vorwissen und Erwartungen

Die erste Studie diente zum einen der Erfassung des Vorwissens von SuS und Sportlehrkräften in Bezug auf die Gefahrenkenntnis im Ballsport an Schulen. Zum anderen sollten Erwartungen der Zielgruppen an die geplante Maßnahme erfasst werden. Insgesamt wurden 92 SuS (33 Mädchen, 59 Jungen, Alter *M* = 13,62, *SD* = 1,54) sowie 9 Sportlehrkräfte (4 Frauen, 5 Männer, Alter *M* = 37,11, *SD* = 6,05) in Form von teilstrukturierten Interviews befragt. Zur Rekrutierung der Stichprobe wurden insgesamt 11 weiterführende Schulen in Baden-Württemberg kontaktiert, wobei drei (Gymnasium, Realschule und Gemeinschaftsschule) die Teilnahme zusagten.

#### Ergebnisse

##### Vorwissen zur Gefahrenkenntnis.

Zur Erfassung des Vorwissens hinsichtlich ballsportspezifischer Unfallmechanismen sollten SuS und Lehrkräfte die Unfallwahrscheinlichkeiten für bestimmte Bewegungen bei den großen vier Ballsportarten anhand von Skalen von 1 (*sehr gering*) bis 5 (*sehr hoch*) einschätzen. Die Ergebnisse in Form der Mittelwerte und Standardabweichungen sind für SuS in Tab. 1 und für Lehrkräfte in Tab. 2 zu sehen. Die Korrektheit der Einschätzungen kann anhand des Vergleichs mit den Unfallmechanismen, die nach Knobloch et al. [11] die höchsten Unfallwahrscheinlichkeiten aufweisen, beurteilt werden. Diese sind in den Tabellen unterstrichen.

**Tab. 1:** Mittelwerte (*M*) und Standardabweichungen (*SD*) für Einschätzungen der Unfallwahrscheinlichkeiten bei Bewegungen in den vier großen Ballsportarten durch SuS (Schülerinnen und Schüler)

Bewegungen	Fußball		Basketball		Handball		Volleyball	
	M	SD	M	SD	M	SD	M	SD
Ballbehandlung ohne Körperkontakt	2,47	1,15	2,05	1,14	2,03	1,16	1,91	0,95
Passen/Fangen	2,26	0,94	2,63	0,97	2,67	1,00	–	–
Absprung	2,27	1,13	2,66	1,11	2,50	0,99	2,57	1,05
Landung	2,52	1,17	**3,09**	1,02	2,75	1,05	2,70	1,06
Laufen ohne Ball	2,01	1,08	1,67	0,89	1,80	1,00	1,75	0,89
Kontakt mit Gegner	**3,88**	0,94	**3,21**	1,01	**3,22**	1,07	2,20	1,23
Von Ball getroffen werden	**3,51**	1,10	**3,79**	1,16	**3,49**	1,22	**3,07**	1,14

*Anm*. *n* = 92. *Unterstrichene Zahlen* kennzeichnen die drei Bewegungen mit den höchsten Unfallanteilen in einer Ballsportart nach Knobloch et al. [11]. *Fett gedruckte Zahlen* kennzeichnen Werte größer oder gleich des Skalenmittelpunkts von 3

**Tab. 2:** Mittelwerte (*M*) und Standardabweichungen (*SD*) für Einschätzungen der Unfallwahrscheinlichkeiten bei Bewegungen in den vier großen Ballsportarten durch Sportlehrkräfte

Bewegungen	Fußball		Basketball		Handball		Volleyball	
	M	SD	M	SD	M	SD	M	SD
Ballbehandlung ohne Körperkontakt	1,89	1,05	1,56	0,53	1,56	0,53	1,89	0,93
Passen/Fangen	2,11	1,05	**3,44**	0,73	**3,22**	0,73	–	–
Absprung	1,67	0,71	2,11	1,27	2,56	1,27	1,89	1,05
Landung	2,44	0,88	**3,67**	0,50	3,44	0,50	**3,00**	0,71
Laufen ohne Ball	2,11	0,93	1,56	0,73	1,78	0,73	1,33	0,50
Kontakt mit Gegner	**4,00**	0,50	**3,44**	0,53	**4,11**	0,53	1,56	1,01
Von Ball getroffen werden	**3,22**	0,83	**3,00**	0,87	**3,33**	0,87	**3,00**	0,71

*Anm. n* = 9. *Unterstrichene Zahlen* kennzeichnen die drei Bewegungen mit den höchsten Unfallanteilen in einer Ballsportart nach Knobloch et al. [11]. *Fett gedruckte Zahlen* kennzeichnen Werte größer oder gleich des Skalenmittelpunkts von 3

Wie aus Tab. 1 ersichtlich, schätzen SuS die Unfallwahrscheinlichkeiten im Fußball größtenteils korrekt ein. Im Basketball (sowie in den anderen beiden Ballsportarten) wurden hingegen die Unfallwahrscheinlichkeiten bei „Ballbehandlung ohne Körperkontakt“, „Passen/Fangen“ und „Laufen ohne Ball“ in Relation zu „Kontakt mit Gegner“ und „Von Ball getroffen werden“ unterschätzt. Lehrkräfte schätzten die Unfallwahrscheinlichkeiten in allen Ballsportarten generell höher ein als SuS (Tab. 2). Dabei entsprachen die Einschätzungen im Vergleich zu denen der SuS eher den realen Unfallwahrscheinlichkeiten. Allerdings wurden auch von Lehrkräften die Risiken bei „Ballbehandlung ohne Körperkontakt“ sowie „Laufen ohne Ball“ tendenziell unterschätzt.

Neben den sportartspezifischen Unfallmechanismen wurden die Teilnehmer*innen auch nach den Unfallwahrscheinlichkeiten bei vertrauten und weniger vertrauten Bewegungen befragt. Hierzu sollten SuS und Lehrkräfte einschätzen, bei welchem Grad der Vertrautheit die größte Unfallwahrscheinlichkeit gegeben ist. Die nach Hübner und Pfitzner [9] risikoreichsten Kategorien von „oft durchgeführten Bewegungen und variablen Bedingungen“ sowie „Wiederholung einer bekannten Bewegung“ schätzten lediglich 32 von 92 SuS korrekt ein. Die Mehrzahl (57) der SuS gab hingegen fälschlicherweise an, dass die Unfallwahrscheinlichkeit bei neu gelernten Bewegungen am höchsten sei. Wie aus der Literatur bekannt, weisen diese jedoch die geringsten Unfallzahlen auf [9].

Im Gegensatz dazu schätzten 6 von 9 Lehrkräften die Unfallwahrscheinlichkeiten für vertraute Bewegungen korrekt ein.

##### Erwartungen an das Präventionskonzept.

Die Fragen nach den Erwartungen der Lehrkräfte betrafen zunächst die inhaltlichen Schwerpunkte, wobei hier koordinative Fähigkeiten, Ballgefühl, Raumwahrnehmung, sowie Unfallmechanismen genannt wurden. Generell erhofften sich die Lehrkräfte von dem „serious game“ Unterstützung im Unterricht durch Motivation der SuS zur Unfallprävention sowie einer Begleitung des Unterrichts durch das Spielen zu Hause. In Bezug auf die didaktischen Begleitmaterialien erwarteten sich Lehrkräfte mehrheitlich klare Erläuterungen, Anleitungen und Regeln zur Durchführung sowie Informationen zu Verletzungen.

### Studie 2 – Spielerleben und Einstellungen

In der zweiten Studie wurde eine vorläufige Version des *Basketball Training Managers* hinsichtlich des Spielerlebens geprüft. Neben 13 SuS (5 Mädchen, 8 Jungen, Alter *M* = 13,11, *SD* = 0,91) nahmen 5 Expert*innen (2 Frauen, 3 Männer, Alter *M* = 34,32, *SD* = 6,22) aus den Bereichen Lehr-Lernforschung sowie Mensch-Computer-Interaktion teil. Zusätzlich wurde die Einstellung von 8 Sportlehrkräften (5 Frauen, 3 Männer, Alter *M* = 40,12, *SD* = 5,01) zum Präventionskonzept bzw. dessen Nutzung erfragt. Die Befragung wurde mittels Online-Fragebögen durchgeführt, wobei die Teilnehmer*innen einen kurzfristigen Zugang zum *Basketball Training Manager* bzw. den Begleitseiten (nur Lehrkräfte) erhielten. Zur Rekrutierung wurden insgesamt 39 weiterführende Schulen kontaktiert, wobei Lehrkräfte von vier Schulen (Gymnasium (2), Realschule und Gemeinschaftsschule) ihre Teilnahme zusagten und ihrerseits SuS kontaktierten. Die Expert*innen wurden aus dem Umfeld der Autor*innen rekrutiert, wobei keine Kolleg*innen der eigenen Abteilungen kontaktiert wurden.

#### Ergebnisse

##### Spielerleben beim *Basketball Training Manager*.

Das Spielerleben der SuS sowie der Expert*innen wurde zunächst mittels des standardisierten Fragebogens GAMEFULQUEST [8] erfasst. Hier sollten die SuS 48 Aussagen anhand von Skalen von 1 (*trifft überhaupt nicht zu*) bis 6 (*trifft voll und ganz zu*) beurteilen. Die Aussagen beziehen sich auf die Faktoren „Leistungsempfinden“ (Bsp.: *Das Spiel regt mich dazu an, meine Leistung hoch zu halten.*), „Herausforderung“ (Bsp.: *Das Spiel fordert mich.*), „Wettbewerb“ (Bsp.: *Das Spiel regt mich zum Wetteifern an.*), „Anleitung“ (Bsp.: *Das Spiel gibt mir das Gefühl, dass mich jemand auf dem richtigen Weg hält.*), „Immersion“ (Bsp.: *Das Spiel fesselt meine ganze Aufmerksamkeit.*) und „Verspieltheit“ (Bsp.: *Das Spiel spricht meine Neugier an.*). Die Ergebnisse sind in Form der Mediane in Tab. 3 abgebildet. Insgesamt empfanden die SuS das Spielen des *Basketball Training Managers* als herausfordernd und leistungsanregend. Außerdem empfanden sie überwiegend eine hohe Immersion beim Spielen. Im Gegensatz dazu war die empfundene Anleitung durch das Spiel gering ausgeprägt. In anschließenden offenen Fragen wurden von den SuS der spielerische Charakter sowie die grafische Gestaltung positiv hervorgehoben. Kritisch angemerkt wurden hingegen fehlende Erläuterungen im Spiel.

**Tab. 3:** Mediane der GAMEFULQUEST Faktoren für SuS (Schülerinnen und Schüler) und Expert*innen

	Leistungsempfinden	Herausforderung	Wettbewerb	Anleitung	Immersion	Verspieltheit
SuS (*n* = 13)	4,38	4,50	4,00	2,29	4,44	4,00
Expert*innen (*n* = 5)	3,44	3,25	4,64	3,57	3,44	4,11

Die Beurteilungen der Expert*innen wurden ebenfalls zunächst mit dem GAMEFULQUEST-Fragebogen erfasst (s. Tab. 3). Dabei fielen die Werte für „Leistungsempfinden“, „Herausforderung“ und „Immersion“ geringer aus als bei den SuS. Im Gegensatz dazu beurteilten die Expert*innen die Faktoren „Wettbewerb“, „Anleitung“ und „Verspieltheit“ höher als die SuS. Bei den offenen Fragen hoben die Expert*innen die grafische Gestaltung des Spiels als positiv hervor. Darüber hinaus wurde der logisch konsistente Aufbau des Spiels als positiv für die Vermittlung der komplexen Sachverhalte betont. Die Kritikpunkte der Expert*innen betrafen ebenfalls fehlende Erläuterungen im Spiel und damit einhergehend eine teilweise erschwerte Verständlichkeit der Spielmechaniken.

##### Einstellungen zum Präventionskonzept.

Die Lehrkräfte wurden mittels offener Fragen zu ihren Einstellungen und Nutzungsabsichten befragt. Die vermittelten Inhalte sowie die dargestellten Übungen wurden von den Lehrkräften dabei überwiegend als nützlich, relevant und geeignet beurteilt. In Bezug auf die zukünftige Nutzung des Präventionskonzepts gaben alle 8 Lehrkräfte an, den SuS das Spiel als „Hausaufgabe“ zu geben und die Inhalte im Präsenzunterricht reflektieren zu wollen. Darüber hinaus konnten sich 6 Lehrkräfte vorstellen, das Spiel zusammen mit den SuS im Unterricht zu spielen. Hierzu ist anzumerken, dass die Befragung im Zeitraum des Distanzunterrichts in Folge der COVID-19-Pandemie durchgeführt wurde, was insbesondere den Sportunterricht erschwerte. Kritisch angemerkt wurde von den Lehrkräften hingegen das Fehlen einer ausführlichen Anleitung zur Nutzung der Spielverwaltung.

## Diskussion und Ausblick

Die bisher durchgeführten begleitenden Evaluationsstudien machen deutlich, dass sowohl der Bedarf als auch die Akzeptanz für das Präventionskonzept bei den Zielgruppen groß ist. In Studie 1 zeigten die SuS z. T. deutliche Defizite in Bezug auf die Gefahrenkenntnis im Ballsport an Schulen. Die in Studie 2 befragten Lehrkräfte bekundeten zudem eine hohe Relevanz der vermittelten Inhalte sowie eine hohe Bereitschaft, das Konzept im Unterricht anzuwenden.

Die Erkenntnisse aus den berichteten Studien dienten darüber hinaus der Entwicklung sowie der Optimierung des Spiels sowie der Begleitmaterialien. In Studie 1 konnte zunächst die Passung zwischen den vermittelten Inhalte und dem Bedarf bei SuS und Lehrkräften bestätigt werden. Die Ergebnisse von Studie 2 dienten hingegen als Basis für konkrete Adaptierungen. So wurden etwa in die finale Version des *Basketball Training Managers *vermehrt Erläuterungen und ein Tutorial implementiert. Zudem wurde in die Begleitmaterialien eine bebilderte Anleitung zur Spielverwaltung eingefügt.

Die Limitationen der vorliegenden Arbeit betreffen vornehmlich die Ad-hoc-Stichproben. Statt durch Zufallsziehungen rekrutierten sich die Proband*innen aus Personen, die womöglich aufgrund eines bestehenden Interesses an einem Präventionskonzept im Blended-learning-Ansatz teilnahmen. Somit ist die Generalisierbarkeit der Studienergebnisse nicht gewährleistet. Darüber hinaus erschwerte in Studie 2 der gewählte Ansatz der Online-Erhebung die Erfassung des Spielerlebens. Die ursprünglich geplante – jedoch durch die Einschränkungen der COVID-19-Pandemie verhinderte – Beobachtungsstudie in Präsenz, hätte ein differenzierteres Bild über das Spielerleben der SuS liefern können. Die zusätzliche Befragung von Expert*innen diente dazu, diese Einschränkung zumindest in Teilen zu kompensieren.

Trotz der angeführten Limitationen lassen die bisherigen Evaluationsergebnisse auf eine hohe Anwendungsbereitschaft des Konzepts seitens der Zielgruppe schließen. Die Prüfung der Wirksamkeit steht hingegen noch aus. Eine entsprechende Evaluationsstudie ist für das 4. Quartal 2021 geplant. Hierbei sollen über einen Zeitraum von mehreren Wochen die Nutzung des Präventionskonzepts an Schulen sowie die Erreichung der kognitiven und motivationalen Lernziele bei SuS geprüft werden.

## Fazit für die Praxis

- Die Gefahrenkenntnis zu Ballsportunfällen an Schulen ist bei Schülerinnen und Schülern (SuS) gering.
- „Serious games“ können bei der zielgruppengerechten Vermittlung kognitiver und motivationaler Lernziele unterstützen.
- Durch den Einsatz in einem Blended-learning-Ansatz können die im Spiel vermittelten Inhalte zur Prävention von Ballsportunfällen in den Präsenzunterricht eingebettet werden.
- Die begleitende Evaluation sicherte den Praxisbezug während des Entwicklungsprozesses.
- SuS und Lehrkräfte zeigen sowohl Bedarf für als auch Akzeptanz gegenüber dem Präventionskonzept.
- Der zukünftige Einsatz des Präventionskonzepts erscheint vielversprechend und prinzipiell auf weitere Unterrichtsszenarien wie Distanzlernen übertragbar.
